# Stigmatization Attitudes and Affecting Factors of Parents with and Without Children with Cancer: A Cross-sectional Study

**DOI:** 10.1007/s13187-025-02581-7

**Published:** 2025-02-18

**Authors:** Aysegul Simsek

**Affiliations:** Department of Pediatric Nursing, Faculty of Health Sciences, Marmara University, BaşibuyukMaltepe, Istanbul, Turkey

**Keywords:** Attitudes, Cancer awareness, Parent’s perspectives, Stigmatization

## Abstract

This study examines whether encountering cancer makes a difference and the perspectives and attitudes of parents of children with cancer. This descriptive and cross-sectional study was conducted with parents who applied to the emergency department of a hospital. Data were collected using a sociodemographic information form and the “Cancer Attitudes Questionnaire (Cancer Stigma)-Community Version.” Mean, minimum, maximum, number, and percentages and comparison analyses (chi-square, Mann-Whitney *U*, and Kruskal-Wallis tests) were performed. The significance level was a 95% confidence interval. The study was completed with a total of 362 parents, 120 of whom had children with cancer. Of the parents, 82.9% are mothers. The scale score of those who had a child with cancer was 3.34 and 3.22 for those who did not have a child with cancer. The scale score was affected by the number of children (*p*=0.008), mother’s occupation (*p*=0.00), parents’ educational status (mother *p*=0.05; father *p*=0.03), family type (*p*=0.00), family economic status (*p*=0.02), religious perception (*p*=0.01), child’s age (*p*=0.001), gender (*p*=0.00), type of treatment (*p*=0.00), and previous hospital experience (*p*=0.006). The findings revealed that parents’ attitudes towards cancer were negative regardless of whether they experienced cancer or not. Personal characteristics such as family type, parental occupation, and educational status, as well as disease-related characteristics such as the type of the child’s disease (acute or chronic) and the type of treatment, affect the direction of attitudes. It is recommended that stigmatization be included in individual and community education on health. Especially for patients, the feelings and effects of stigmatization can be included. It is also recommended to include an empathic approach in education.

## Introduction

Human behaviors have a great impact on the emergence of psychosocial problems. Stigma, one of these behaviors, is a process that leads to discrimination as a result of labeling and stereotyping [1–3]. Health-related stigma is often related to living with a specific disease or health problem [4–7]. In some cases, stigma can be as dangerous as the disease itself [8]. Children diagnosed with cancer and their families complain of stigmatization in addition to the disease [9]. When stigmatization occurs, the social identity of the child and his/her parents is damaged and this negatively affects the child’s well-being [10, 11]. Quality of life and even life expectancy decrease due to decreased treatment adherence, increased frequency of symptoms, and negative effects of treatment [12, 13]. Socially imposed stigma is characterized by thoughts and beliefs that cancer is a fatal and incurable disease and that patients who recover are unfit on physical, mental, and social levels. Cancer is seen as a social problem due to the social and economic cost it imposes on society, which contributes to negative attitudes and perceptions about the condition [14].

In the literature, there are many studies examining the relationship between chronic diseases and health problems and stigmatization in adults. These are often psychological problems such as schizophrenia and autism [12, 13, 15, 16]. However, no study has been found to investigate the thoughts and attitudes of parents towards a life-threatening condition such as cancer, especially the thoughts and attitudes of parents with children with cancer towards stigmatization. The perception and adaptation to serious illness in adulthood are thought to be easier than in children. The situation is different when it comes to children during the growth and development period. It is difficult for children to understand and adapt to the concepts of health and illness according to their age. It is also thought that stigmatization experienced in childhood may have negative effects in adulthood. The same is true for parents who are responsible for the care of their children. A serious illness in their child, compared to a problem in themselves, affects their adaptation as well. In such a situation, experiencing stigmatization may make coping and treatment more difficult. There are no similar studies in the literature to support this situation. Based on this, we aimed to determine the perspectives and attitudes of parents who have children with cancer and whether facing cancer makes a difference. Secondarily, it was aimed to determine whether there is a difference in cancer stigmatization for these parents compared to the parents with children without cancer diagnosis.

## Methods

The study is a descriptive, cross-sectional, comparative study. In the study, the perspectives and attitudes towards the stigmatization of parents with and without cancer were compared. The study population consisted of parents of children who presented to the emergency department between May 2022 and July 2024. The uncertainty associated with an acute symptom, especially the idea that it is caused by a serious illness (such as cancer), affects parents’ perspectives. It was thought that this may have an effect on measuring the presence of a behavior such as stigmatization. Therefore, parents who brought their sick child to the emergency department were included. The sample size was determined as 145 people with a 95% confidence interval of 0.80 (80%), an effect size of 0.50, and *α*=0.05 by power analysis. The study was completed with 362 parents. Parents with cognitive or neurological impairment, communication problems, and incomplete completion of the study questions were excluded from the study. The Equator Network - Strobe checklist was used for reporting the article.

A sociodemographic information form was used to question the characteristics of the parents and their children, and the Questionnaire for Measuring Attitudes Toward Cancer (Cancer Stigma)—Community Version (MATCQ) was used to measure attitudes towards cancer [3, 4, 7, 12, 17]. A score above 2.5 on the scale indicates an increase in negative attitudes towards cancer. While the overall Cronbach’s alpha value of the scale was 0.89, it was calculated as 0.91 in our study.

Data were obtained from parents of children whose condition stabilized after presentation to the emergency department. Parents who met the inclusion criteria were informed about the study. They were told that questions would be asked at their convenience. After this information, all parents agreed to participate in the study. The data were collected by the researchers in the interview room in the emergency room by face-to-face interview method in an average of 10–15 min. Data were collected by interviewing the parents one time. The study was completed with 362 parents, 300 mothers (82.9%) and 62 fathers (17.1%).

Data were analyzed in a statistical analysis program. In the first stage, descriptive statistics were calculated for the distribution of the characteristics of the sample group. After the normality distribution was determined by the Kolmogorov-Smirnov test, Mann-Whitney *U*, Kruskal-Wallis test, and one-way ANOVA tests were used for comparison analysis. Simple regression analysis was used to determine the factors predicting MATCQ scale score. *p*<0.05 was accepted as a 95% confidence interval.

Ethics committee permission (dated 10.02.2022 and numbered 2022/33) was obtained before the study. Written and verbal informed consent of the parents was also obtained. The Helsinki Declaration of Human Rights was complied with in the study.

## Results

**Fig. 1:**
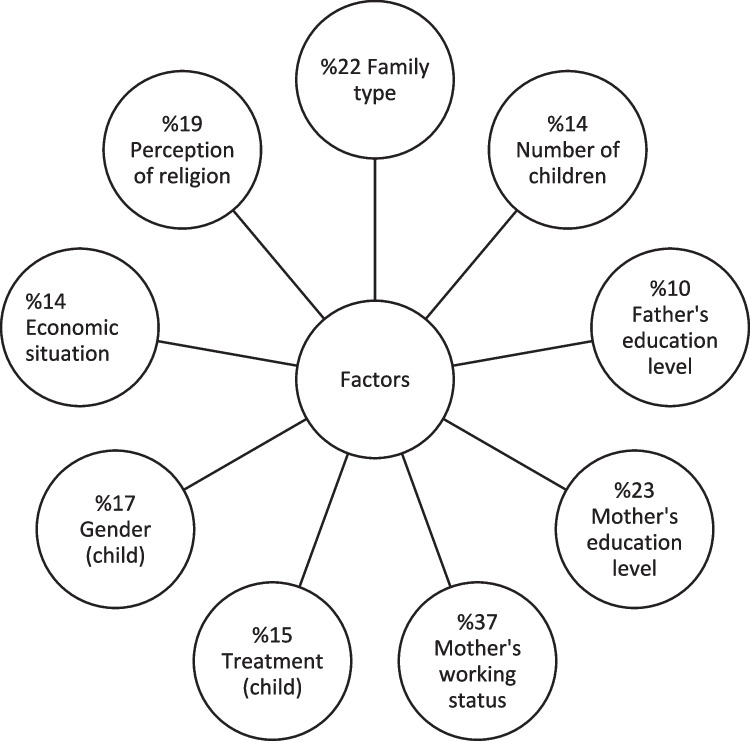
Prediction of the factors affecting the score of the MATCQ (MATCQ: The Measuring Attitudes Towards Cancer Questionnaire—Society Version; Constructed according to the results of simple regression analysis)

The study was completed with 362 parents, 120 of whom had children with cancer and 242 of whom had children with acute illness. Table 1 shows the mean MATCQ total and subscale scores of the parents with an average age of 30–34 years. The distribution of family characteristics and descriptive information about the parents are presented in Table 2, and family-related factors affecting MATCQ score are shown in Table 3. In addition, the factors predicting the MATCQ scale score are shown in Figure 1.

**Table 1:** Distribution of total and subscale mean scores of the Questionnaire for Measuring Attitudes Towards Cancer (Cancer Stigma)-Community Version

Scale		Acute Disease		Cancer		*p*
		Mean±Sd	Min-max (med)	Mean±Sd	Min-max (med)	
MATCQ	Cancer detection, dissemination	3.04±0.83	1–4 (3)	3.3±0.8	1–4 (3.2)	*0.003
	Impossibility of recovery	3.17±0.62	1–4 (3)	3.02±0.7	1–4 (3)	*0.049
	Discrimination	3.36±0,72	1–4 (3)	3.55±0.64	1–4 (4)	*0.014
	Total	3.22±0.65	1–4 (3)	3.34±0.69	1–4 (3.5)	*0.071

*Sd*, Standard deviation; *min*, minimum; *max*, maximum; *med*, median; *MATCQ*, The Measuring Attitudes Towards Cancer Questionnaire—Society Version

^*^Mann-Whitney *U* test

*p*<0.05

**Table 2:** Distribution of family characteristics according to the disease characteristics of their children

Features			Acute disease		Cancer		*p*
			Mean±Sd	Min-max (med)	Mean±Sd	Min-max (med)	
Parent	Number of hospitalizations		1.8±0.7	1–5(2)	3.9±4.5	1–31(1)	*0.000
	Age of mother		30.1±7.2	21–58(28)	34.7±8.1	21–54(33)	*0.000
	Father’s age		33.2±6.9	24–60(32)	37.4±6.7	28–58(36)	*0.000
	Number of children		2±1.1	1–5(2)	2.3±0.8	1–6(2)	*0.005
			n	%	n	%	
	Mother’s working status	Not working	197	81.4	87	72.5	*0.058
		Working	45	18.6	33	27.5	
	Mother’s education level	Elementary	66	27.4	36	30.1	**0.600
		Middle-school	62	25.6	31	25.8	
		High school	64	26.4	30	25	
		University	50	20.6	23	19.1	
	Father’s working status	Not working	2	0.8	0	0	*0.344
		Working	240	99.2	112	100	
	Father’s education level	Elementary	56	23.3	26	21.7	**0.907
		Middle-school	56	23.1	30	25	
		High school	72	29.7	34	28.3	
		University	58	23.9	30	25	
	Family type	Nuclear family	195	80.6	106	88.3	*0.041
		Large family	47	19.4	14	11.7	
	Economic situation	Income less than expenditure	104	42.9	32	26.6	**0.001
		Income equal to expenditure	114	47.1	66	55	
		Income more than expenditure	24	10	22	18.4	
	Perception of religion	I believe. I fulfill all its requirements	167	69	90	75	*0.269
		I believe. I fulfill some of its requirements	75	31	30	25	
Children	Previous hospital experience	No	38	15.8	34	28.4	**0.000
		Yes	204	84.2	86	71.6	
	Age	Newborn	9	3.9	0	0	**0.000
		1 month–1 year	63	26	6	5.1	
		2–3 years old	72	29.7	30	25	
		4–6 years old	56	23.1	47	39.1	
		7–12 years old	26	10.7	13	10.8	
		≥13 years old	16	6.6	24	20	
	Gender	Girl	111	45.9	63	52.5	***0.264
		Boy	131	54.1	57	47.5	
	Status of school attendance	No	197	81.4	54	45	***0.000
		Yes	45	18.6	66	55	
	Treatment	CT	0	0	62	51.6	**0.000
		RT	0	0	3	2.7	
		Surgery and CT	0	0	33	27.5	
		Surgery	10	4.2	5	4.1	
		Surgery and RT	0	0	4	3.3	
		Antibiotics	24	9.9	13	10.8	
		Other	208	85.9	0	0	
	Total		242	100	120	100	

*Sd*, Standard deviation; *min*, minimum; *max*, maximum; *med*, median; *n*, number; *%*, percentage; *CT*, chemotherapy; *RT*, radiotherapy

^*^Chi-square test

^**^Kruskal-Wallis test

^***^Mann-Whitney *U* test

*p*<0.05

**Table 3:** Family-related factors affecting the score on the Questionnaire for Measuring Attitudes Toward Cancer (Cancer Stigma)-Community Version

Features			Med (min-max)	MATCQ
Parents	Number of hospitalizations		2 (1–16)	*0.906
	Age of mother		30 (21–58)	*0.852
	Father’s age		34 (24–60)	*0.285
	Number of children		2 (1–6)	*0.008
			*n* (%)	
	Mother’s working status	Not working	284 (78.5)	**0.000
		Working	78 (21.5)	
	Mother’s education level	Elementary	102 (28.2)	***0.000
		Middle school	93 (25.7)	
		High school	94 (26.0)	
		University	73 (20.2)	
	Father’s education level	Elementary	82 (22.6)	***0.003
		Middle school	86 (23.8)	
		High school	106 (29.3)	
		University	88 (24.3)	
	Family type	Nuclear family	301 (83.2)	**0.000
		Large family	61 (16.9)	
	Economic situation	Income less than expenditure	136 (37.6)	***0.002
		Income equal to expenditure	180 (49.7)	
		Income more than expenditure	46 (12.7)	
	Perception of religion	I believe. I fulfill all its requirements	257 (71)	*0.001
		I believe, I fulfill some of its requirements	105 (29)	
Children	Medical diagnosis	Akut diseases	242 (66.9)	**0.071
		Cancer	120 (33.1)	
	Previous hospital experience	No	72 (19.9)	**0.006
		Yes	290 (80.1)	
	Age	Newborn	9 (2.5)	***0.001
		1 month–1 year	69 (19.1)	
		2–3 years old	102 (28.2)	
		4–6 years old	103 (28.5)	
		7–12 years old	39 (10.8)	
		≥13 years old	40 (11)	
	Gender	Girl	174 (48.1)	**0.000
		Boy	188 (51.9)	
	School attendance status	No	251 (69.3)	**0.188
		Yes	111 (30.7)	
	Treatment	CT	62 (17.1)	***0.000
		RT	3 (0.8)	
		Surgery and CT	33 (9.1)	
		Surgery	15 (4.1)	
		Surgery and RT	4 (1.1)	
		Antibiotics	37 (10.2)	
		Other	208 (57.5)	

*min*, minimum; *max*, maximum; *med*, median; *n*, number; *%*, percentage; *CT*, chemotherapy; *RT*, radiotherapy; *MATCQ*, The Measuring Attitudes Towards Cancer Questionnaire—Society Version

^*^Chi-square test

^**^Mann-Whitney *U* test

^***^Kruskal-Wallis test

*p*<0.05

## Discussion

The study, which aimed to determine the perspectives and attitudes of parents with and without cancer towards stigmatization, was completed with 362 parents. Well-being includes both physical and psychosocial well-being [18]. The presence of illness in children affects social needs such as family processes, coping mechanisms, socialization, and communication. The perception of illness is influenced by individual or environmental factors as well as what society thinks (stigmatization behaviors, etc.). Although stigmatization seems to be directed towards the child and family, it is also a social problem [5, 15, 19, 20]. In other words, whether their own children have cancer or not, cancer has evoked negative feelings and thoughts in these parents. In the study, it was determined that the acute illness group scored an average of 3.22 points on the MATCQ, while the cancer group scored an average of 3.4 points, and parents had negative attitudes towards cancer. Our study findings are similar to the literature. That is to say, in one study, it was reported that most of the sample thought that cancer was incurable and prevented socialization [17]. The same scale was administered to 301 participants whose mean age was similar to that of the present study. It was reported that the total score of the scale was low, but the score of the impossibility of recovery sub-dimension was high [15]. It can be said that psychosocial behaviors and attitudes affect not only their own well-being but also the well-being of others and may negatively affect recovery in cancer patients.

In cancer stigmatization, reactions to the disease are influenced by multiple factors. These are usually factors related to the parent, the child, and the nature of the disease. The findings obtained in our study also confirm this. In a study comparing gender in the MATCQ discrimination subscale, it was reported that men’s attitudes towards cancer stigmatization were negative, and there was no difference between parental age and economic status and scale score [15]. In a study investigating the adaptation of 111 families of children with cancer to the disease, it was reported that the severity of the disease negatively affected adaptation, while the presence of communication and support positively affected it [19]. This confirms that reactions to the disease are affected by individual and disease-related factors.

Parents’ level of education is important in understanding the nature of the disease and coping with it. In our study, the educational level of mothers in the cancer group was higher. Fathers were high school graduates in both groups. Although the scale score was low, there was a significant difference between the level of education and the impossibility of recovery subscale. In a similar study, it was reported that the educational level was mostly at the primary school level, but the MATCQ scale score was low [19]. In line with these studies, it can be said that education is an important factor. These findings suggest that education is an important and positive factor in understanding the diagnosis and treatment processes, perception of the disease, and use of coping mechanisms.

In our study, it was determined that the parent who received information and was present with the child was mostly the mother with 82.9%. This confirms that the “mother is the primary responsible for child care” from past to present. The father’s role of meeting the basic needs in the first step of Maslow’s hierarchy of basic needs has not changed [21]. Thus, while maintaining this role, the father also assumes the roles of illness and care. Moreover, the predominance of masculine behaviors in our society and the fact that the role of childcare is thought to be in the mother makes this situation even more difficult [22]. This shows which parent is the primary caregiver and how different roles they have affect attitudes towards cancer. Therefore, learning the psychosocial attitudes, thoughts, and experiences of parents towards the disease, whether acute or chronic, and developing evidence-based interventions for negativities will prevent the deterioration of family processes and make it easier to cope with the disease.

In our study, 66.9% of the children came to the hospital with symptoms of an acute illness and 33.1% with a complication related to cancer. The type of disease affects the patient and parents differently. The presence or absence of acute or chronic illness or treatment affects the mental state of parents as well as their daily lives. In cultures where family members undertake patient care, as in our country, religion and cultural practices play a vital role in guiding patient care [22]. People often turn to religion as a coping mechanism when their child, themselves, or their family has an illness. In the present study, the perception of religion in both groups was mostly “I believe in it, I fulfill all its requirements.” Parents turn to spirituality due to lack of social support, fear, or experience of stigmatization and exclusion. Anti-cancer stigma programs health professionals should provide religious and culturally sensitive care and adopt a family-centered care approach.

The information obtained from the present study shows that parents’ thoughts, attitudes, and behaviors are affected according to the type of disease. When it comes to their child, parents’ reactions may vary in each of the first or repeated hospitalizations and admissions. Reactions when diagnosed with a curable disease differ when diagnosed with cancer. This is due to the association of cancer with the concepts of death and continuous hospitalization and treatment. It was observed that every parent in the present study had a negative attitude towards cancer, whether or not their child was diagnosed with cancer. When considered, it was observed that stigmatization may develop towards those who have a disease, whether they have cancer or not. With education, stigmatization and its effects on the patient and his/her family can be made known to the society. Education can raise awareness and prevent stigmatization.

The lack of a similar study is a limitation. The fact that the result obtained from the study was contrary to what was thought (even those with children with cancer had high cancer stigmatization scores) is a strength.

## Conclusion

In conclusion, cancer stigmatization negatively affects coping mechanisms for the child and family seeking support. Similarly, it also affects the healing process. It was a surprising finding that cancer stigmatization was high even among parents whose children had cancer.

Considering the findings on cancer stigmatization in this article, it may be recommended to include information and trainings on stigmatization and discrimination in the scope of cancer awareness activities. Thus, the psychosocial burden experienced by the family struggling with cancer can be alleviated, and social support can be provided. It should not be forgotten that social well-being also affects physical well-being. It may be recommended that health professionals plan a child and family-centered care and biopsychosocial framework of care within the framework of available evidence.

## Data Availability

Data is available on request.
